# [Retracted] miR-29a inhibits human retinoblastoma progression by targeting STAT3

**DOI:** 10.3892/or.2026.9207

**Published:** 2026-10-08

**Authors:** Shu Liu, Xiaomeng Zhang, Chunmei Hu, Yingxue Wang, Chunling Xu

Oncol Rep 39: 739–746, 2018; DOI: 10.3892/or.2017.6144

Following the publication of this paper, and a corrigendum (doi: 10.3892/or.2017.6144) published in 2021 to take account of duplicated western blot data that were identified in Fig. 4D, it has subsequently come to light that flow cytometric plots featured in Fig. 2D and Transwell invasion assay data in Fig. 3B, both on p. 742, contained data that were strikingly similar to data that had already been published in articles written by different research groups at different research institutes in a number of different journals.

Given that the contentious data named above had already been published prior to the receipt of this paper at *Oncology Reports*, the Editor has decided that this paper should now be retracted from the Journal. After contacting the authors, they accepted the decision to retract the paper. The Editor apologizes to the readership for any inconvenience caused.

